# Avoiding future controversies in the Alzheimer’s disease space through understanding the aducanumab data and FDA review

**DOI:** 10.1186/s13195-023-01238-1

**Published:** 2023-05-24

**Authors:** Samuel P. Dickson, Sean Hennessey, Jessie Nicodemus Johnson, Newman Knowlton, Suzanne B. Hendrix

**Affiliations:** Pentara Corporation, Millcreek, UT USA

**Keywords:** Alzheimer’s disease, Clinical trials

## Abstract

Key points of disagreement between the aducanumab FDA statistical review, which had primarily negative conclusions, and the clinical review, which had primarily positive conclusions, were investigated. Results from secondary endpoints in positive Study 302 were significant and these endpoints provided meaningful additional information. Findings indicate the statistical review of the aducanumab data was incorrect in a number of key areas. Greater placebo decline was not responsible for the significant results in Study 302. Correlations did exist between reduction in β-amyloid and clinical outcomes. Missing data and functional unblinding did not likely bias results. In contrast, the clinical review went too far in saying the negative results in Study 301 did not detract from the positive results in Study 302, as all clinical data should be considered in the evaluation, and the clinical review accepted the company’s explanation for divergence of the results between the studies although much of the divergence remained unexplained. Interestingly, both the statistical review and the clinical review considered the available efficacy evidence despite both studies being terminated early. Implications of these findings include that the divergence in results seen in the two phase 3 aducanumab studies can be expected in other studies with similar design and analysis. Therefore, further research is needed to determine if analysis methods other than MMRM and/or optimized outcomes will provide more consistent results across studies.

**Supplementary Information:**

The online version contains supplementary material available at 10.1186/s13195-023-01238-1.

## Introduction

The aducanumab approval process has been uncharacteristically controversial. At the core of the controversy are the discrepancies between the clinical and statistical reviews and the interpretation of those reviews by the advisory committee. While the topic remains controversial, significant polarization and often dogmatic opinions arose at the time of the advisory committee meeting and subsequent approval that interfered with a fair evaluation of the evidence and arrival at rational conclusions. While this polarization is clear in the uproar across the media, this dogma originated at the advisory committee meeting and was primarily driven by the widely disparate clinical and statistical reviews. With time and recent events, including the successful trial of a different monoclonal antibody targeting Aβ (lecanemab), a more objective evaluation of the evidence for and against the efficacy of aducanumab may now be possible. With this in mind, we reconcile the differences between the two key reviews in this article.

Alzheimer’s disease (AD) is a complex and heterogeneous neurodegenerative disease. Our ability to develop novel treatments for AD depends on our ability to accurately diagnose patients, to accurately measure individuals’ disease severity, and to reliably analyze the data.

All three requirements pose significant challenges and can therefore lead to difficulties in clear interpretation of the data. The Phase 3 clinical trials of aducanumab illustrate these interpretive challenges [1]. The efficacy and safety of aducanumab was assessed via Study 301, ENGAGE, and Study 302, EMERGE, two identically designed, phase 3, randomized clinical trials in patients with early AD. Study 301 failed to meet its primary objective, whereas Study 302 met its primary objective. Aducanumab was granted accelerated approval by FDA based on its ability to reduce a defining pathophysiological feature of Alzheimer’s disease, Aβ plaques, and the determination that this reduction was predictive of clinical benefit [2, 3].

Publicly available documents show that the results from the aducanumab clinical program were viewed as supporting full approval by the clinical review team at FDA, but not the statistical review team [4]. Not surprisingly, the results have attracted disparate interpretations along with positive and negative attention within and outside of the Alzheimer’s research community [5–19]. Although many of these sources offered opinions on the unusual circumstances and process leading up to the approval of aducanumab, what has been lacking is a detailed examination of the results that led to the different interpretations within FDA.

Therefore, the purpose of the present investigation is to review the key points of disagreement between the statistical review and the clinical review at FDA and to review the key results that fostered those different opinions with a statistical perspective. It is hoped that reviewing these issues and data now will help set the stage for more straightforward interpretations of results from future trials because, regardless of one’s opinion on the approval of aducanumab, the controversy surrounding the results has benefitted no one and has interfered with continued development of effective treatments.

## Methods

This investigation was conducted on publicly available information without access to the raw data. It began with a detailed review of the aducanumab FDA Advisory Committee briefing materials, including the Advisory Committee briefing book [4], which also contained the clinical and statistical reviews as appendices. In addition, sponsor and FDA presentations at the Advisory Committee meeting and the transcripts of those presentations were reviewed [20–24]. This review identified key areas of disagreement between the clinical and statistical review teams at FDA that were cited by Advisory Committee members when justifying their votes regarding the questions posed to them by FDA. For each of these key areas of disagreement, the same documents were reviewed, and the rationale and results used to support the opposing positions were summarized.

The two general areas of disagreement were (1) robustness of the positive result in Study 302 and (2) the degree to which the Study 301 results detracted from (contradicted) the Study 302 findings. Regarding the robustness of Study 302, the following specific questions were identified: (a) Were results from the secondary endpoints statistically significant, and if so, did they provide important additional information? (b) Were reductions in amyloid beta related to changes in clinical outcomes? (c) Did differences in the magnitude of placebo decline influence differences in results? (d) Did missing data compromise the validity of the results? and (e) Did functional unblinding compromise the validity of the results?

A global statistical test (GST) was performed on the publicly available analysis summaries [1] to provide integrated evidence of efficacy across outcomes within each study and across studies. The GST is performed using the following equation:

$$Z= \frac{\overline{z} }{\sqrt{\frac{1+\rho \left(k-1\right)}{k}}}$$

where $\overline{z }$ is the mean of the z-scores of the test of each treatment difference across outcomes, $k$ is the number of outcomes being combined, and $\rho$ is the average pairwise correlation between outcomes. Within the study, $\rho$ was assumed to be 0.3, while between studies $\rho$ was assumed to be 0.

## Results

EMERGE and ENGAGE were two identically designed, randomized, double-blind, placebo-controlled, global, phase 3 studies of aducanumab in patients with early Alzheimer’s disease. A total of 3285 patients were enrolled at 348 sites in 20 countries [1638 (EMERGE) and 1647 (ENGAGE)], 1812 (55.2%) of which completed the study. ENGAGE (first treatment: August 13, 2015) started 1 month earlier than EMERGE (first treatment: September 15, 2015); recruitment for both ended in July 2018. No obvious imbalances in baseline demographics were noted across treatment arms. The studies were completed at week 78 (December 26, 2018), but due to early termination of the studies, the median postbaseline visit occurred at 13.6 months (range, 9.5 to 19.6 months).

Throughout the statistical review [4] (Appendix II) concerns were raised regarding the study design and data quality suggesting that Study 302 was no longer an adequate and well-controlled study with a positive outcome. In contrast, the clinical review stated: “The effect of aducanumab in Study 302 was robust, including convincing effects on the primary endpoint, all three multiplicity‐controlled secondary endpoints, and the tertiary clinical endpoint, and was exceptionally persuasive on several of the instruments used to evaluate efficacy.” ([4], pg. 148). Quoting statements made in the statistical review, the Advisory Committee vote did not support that Study 302, viewed independently, provided strong evidence for the effectiveness of aducanumab, although four of the members with “no” votes and one a non-voting member specifically mentioned that Study 301 could not be ignored (see Supplement Sect. 1).

Before reviewing the data in detail, it is relevant to note key regulatory interactions between the sponsor and FDA. In 2015, the sponsor obtained Special Protocol Assessment (SPA) agreements from the FDA for the Phase 3 studies. FDA agreed that the design and planned analysis of each Phase 3 study addressed the objectives necessary to support a regulatory submission. In addition, the sponsor and FDA had three Type C meetings prior to the Advisory Committee meeting (June 2019, October 2019, and February 2020) at which the design and data concerns indicated below were reviewed and discussed ([4], pg. 19). Information from these interactions is critical to understanding the aducanumab submission.

### Statistical significance of secondary endpoints

The statistical review concluded that none of the secondary endpoints for the high-dose group of Study 302 was significant because the low dose was not significant for the primary endpoint ([4], pg. 255), thereby halting the sequential testing procedure. In contrast, the clinical review considered all three secondary endpoints to be statistically significant ([4], pg. 148).

The evidence reviewed for this investigation showed that the multiplicity adjustment approach used in Studies 301 and 302 was prespecified in the study protocols to cover multiple doses and multiple endpoints and was part of the SPA agreement. The key principle of the multiplicity approach used in Studies 301 and 302 was that failure of low dose should not stop testing for high dose.

The primary endpoint of CDR-SB high dose was significant (*p* = 0.0120). Testing simultaneously moved to high-dose MMSE and low-dose CDR-SB. MMSE high dose was significant (*p* = 0.0493). Low-dose CDR-SB was not significant *p* = 0.0901). Therefore, all subsequent low-dose tests were not significant. However, testing for high dose continued, as per the protocol specified procedure, because significance of CDR-SB for the low dose was not required to proceed with testing high-dose secondary endpoints. Testing proceeded to high-dose ADAS-Cog13, which was significant (*p* = 0.0097), and then to high-dose ADCS-ADL-MCI, which also was significant (*p* = 0.0006).

The statistics review misinterpreted the multiplicity adjustment scheme, failing to appreciate that after significance on the primary outcome, high-dose CDR-SB, testing proceeded to low-dose CDR-SB *AND* high-dose MMSE. This testing scheme is not as common as a purely sequential approach anticipated by the statistical review. However, the approach was pre-specified and agreed to by FDA. Hence the secondary endpoints were significant, adding to the already compelling evidence from the primary endpoint in Study 302. It is also worth noting that it would be highly unusual to require significance on a low dose as a gatekeeping test to allow evaluation of secondary endpoints for a high dose that achieved significance on a primary endpoint when the high dose was clearly prespecified to be prioritized over the low dose.

### Importance of secondary endpoints

The statistical review concluded that “the four key endpoints do not measure very distinct efficacy information, i.e., one or at most 2 captures the key information” ([4], pg. 338) and used this conclusion to support the use of the CDR-SB alone to derive conclusions, not considering the secondary endpoint results. In contrast, the clinical review stated results to be “… exceptionally persuasive on several of the instruments used to evaluate efficacy” ([4], (pg. 148) thereby implying that they believed the secondary endpoints were providing important additional information. The clinical review further stated: “FDA routinely encounters the use of these measures and they are appropriate selections for use in supporting an effect on an acceptable primary measure. The principal components analysis indicated that while there may be overlap among the 4 clinical endpoints, each also captures distinct information regarding cognitive decline. Effects on each of these endpoints can independently contribute to the persuasiveness of a specific study” ([4], pg. 167).

**Fig. 1:**
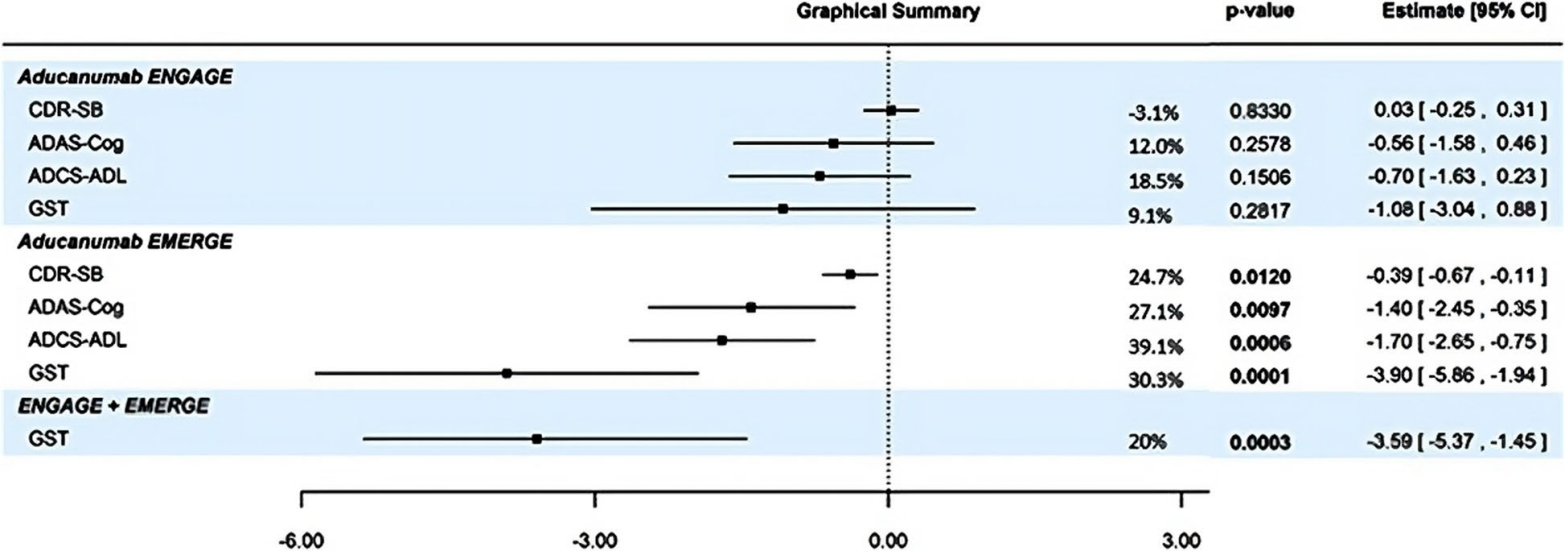
Forest plot of key endpoints in Study 301 and Study 302 with GSTs combining endpoints within the studies and between studies. The assumed correlation between endpoints within studies was 0.3, while correlation between studies was assumed to be 0 due to the independent populations

The statistical review assessment was based on a principal component analysis that demonstrated that 64% of the information was captured using the first principal component. Although it is true that a single principal component accounts for a little more than 60% of the information in the four endpoints, the primary endpoint—CDR-SB—does not comprise this first principal component. A GST combining all 4 endpoints aligns better with this principal component and shows much different results than the CDR-SB alone with an effect size of 9.1% slowing (*p* = 0.2817) in Study 301 (ENGAGE) and 30.3% slowing (*p* = 0.0001) in Study 302 (EMERGE). An integrated analysis across both studies using summary data resulted in 20% slowing and *p* = 0.0003 (see Fig. 1).

Moreover, if the 4 scales measured similar aspects of AD, and provided no independent information, then it is unlikely that results would have differed so much from one scale to another, with slowing of decline relative to placebo of 18% on the MMSE but 40% on the ADCS-ADL-MCI as reported in the briefing book. The fact that the first principal component explains only 64% of the variance across endpoints demonstrates the unique contributions of these items. The correlations between CDR-SB, ADAS-Cog, and ADCS-ADL change scores at 18 months vary between 0.30 and 0.60. Since the square of the correlation is the percent of overlapping information (variance), the scales overlap between 9 and 36% with each other, demonstrating the distinct efficacy information.

Historical 18-month studies in similar populations have observed correlations of approximately 0.50, with r-squared values of less than 0.25 indicating that less than 25% of the information in one scale is captured by another scale. These results suggest that the 4 primary and secondary endpoints measure distinct aspects of cognition and function. The consistent efficacy observed across these diverse factors aligns with the Clinical reviewer’s interpretation that statistical significance on all three secondary endpoints is a much more persuasive finding than significance on the primary outcome alone.

### Association between changes in amyloid beta and changes in clinical outcomes

The statistical review concluded that there was no correlation between changes in β-amyloid and changes in clinical outcomes ([4], pg. 294) and this conclusion was stated multiple times by the Advisory Committee members during the Advisory Committee meeting. The clinical review concluded that significant but relatively weak correlations existed. The clinical review further noted that the simple correlations did not account for a number of potentially prognostic factors nor the time lag between β-amyloid and slowing of decline on clinical outcomes. The clinical review further noted that an exposure response model detected association between reduction of β-amyloid and slowing of decline on clinical outcomes ([4], pg. 183).

An important consideration that was not explicitly addressed in the advisory committee meeting was the distinction between patient-level and group-level correlations. Patient-level correlations assess whether individual patient changes in β-amyloid are associated with changes in clinical outcomes (higher with higher and lower with lower) and are relevant in understanding to what degree response on the biomarker predicts response on clinical outcomes for individual patients. The group-level correlations, which assess whether interventions with the larger average change in β-amyloid are associated with a better clinical response, can provide additional insight and are relevant in understanding to what degree mean response on the biomarker predicts mean response on clinical outcomes in a group of patients, such as the dose groups that were formed by randomization.

The statistical review based its conclusion on patient-level correlations only, using an analysis of a subset of data, without accounting for key baseline factors or considering results from the exposure–response model. The conclusion from the clinical review utilized patient-level and group-level correlations based on all the data, with correlations that accounted for baseline factors, so the clinical review’s handling of correlation demonstrated greater sophistication and accuracy and is more likely to lead to correct interpretation.

**Fig. 2:**
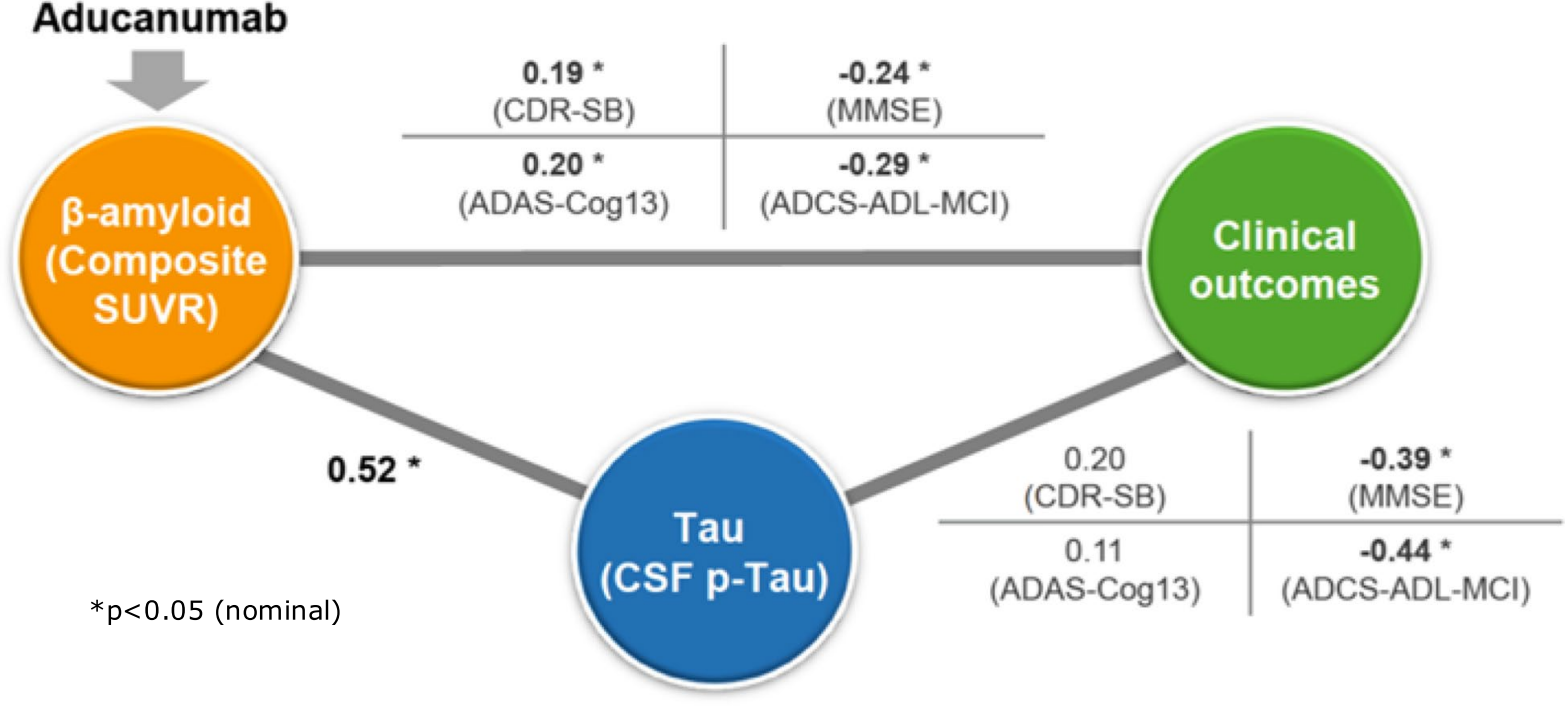
Aducanumab-related biomarker changes are associated with slowing in clinical decline (Study 302). Correlations between biomarkers and clinical outcomes. All associations are partial Spearman correlation of change from baseline to week 78 between each variable

Figure 2 summarizes patient-level correlations between biomarkers and clinical outcomes from sponsor slides based on analyses that included all data and accounted for baseline factors ([20], slide 55) and achieved statistical significance for the primary and all 3 secondary outcomes.

**Fig. 3:**
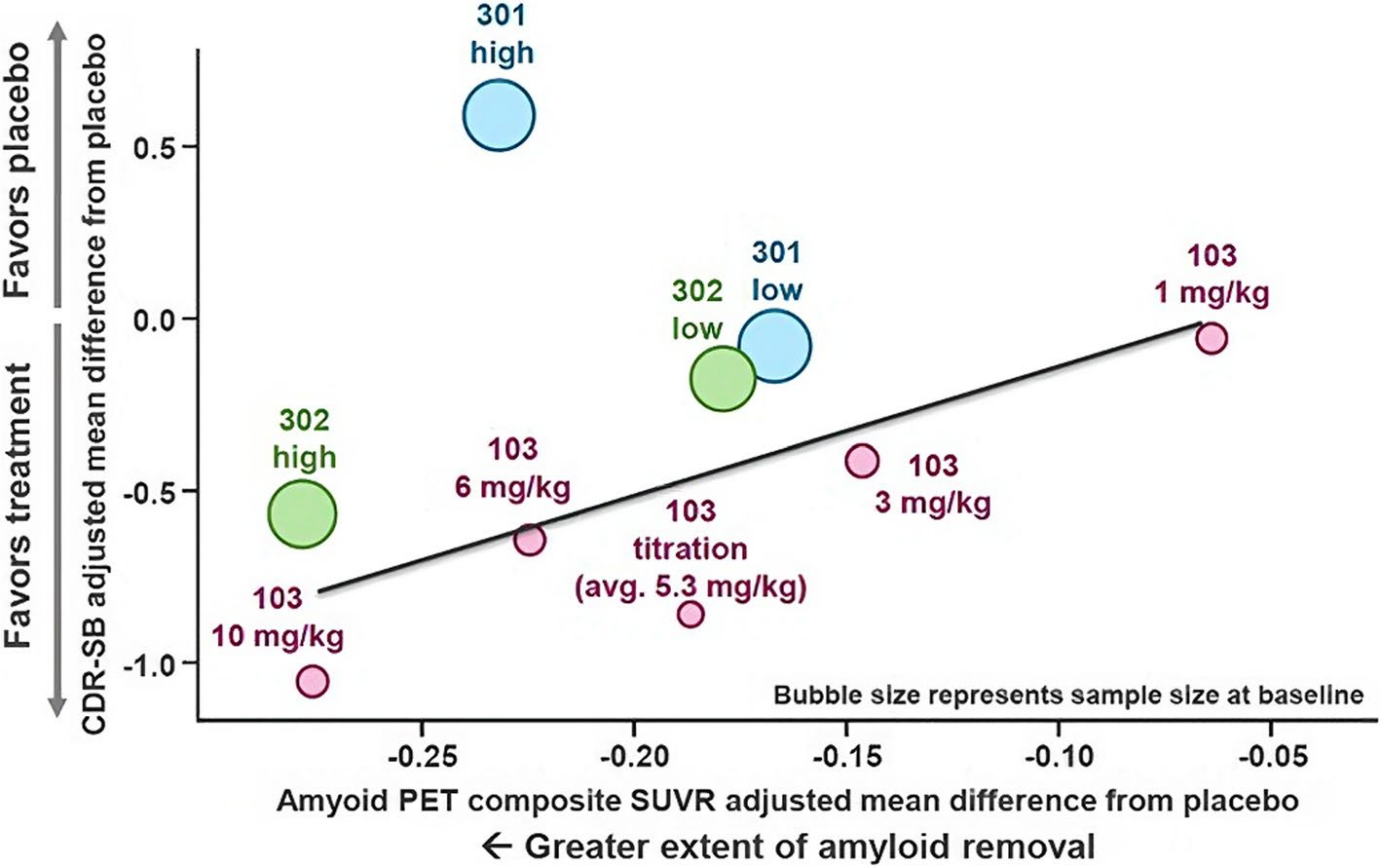
Study 301 high-dose group diverged from an otherwise consistent association between Aβ reduction and slowing of clinical decline. Associations between changes in Aβ and changes in CDR-SB by randomized treatment groups in Studies 103, 301, and 302

Figure 3 summarizes the group-level correlations by showing changes from baseline in the 9 active treatment groups across the two phase 3 and proof-of-concept studies for CDR-SB and β-amyloid ([20], slide 58). These results show (1) a dose–response relationship for reduction in β-amyloid; (2) a dose–response relationship for slowing of decline in CDR-SB; (3) a consistent group-level association between reduction in β-amyloid and slowing of decline in CDR-SB, except for; and (4) the outlier results on CDR-SB from the high dose of Study 301.

These group-level results are consistent with a dose-exposure–response relationship for Aβ plaque reduction (SUVR) and clinical outcomes. Correlations between Aβ plaque reduction and clinical outcomes were significant, but not sufficiently strong to be useful predictors of individual patient outcomes, but were strong for group-level correlations, consistent with expectations for a relationship between an upstream biomarker and clinical outcomes.

### Influence of placebo decline on efficacy results

Placebo decline is important to assess because it is not possible to demonstrate a slowing of decline if there is no decline. The statistical review stated: (1) “A worse placebo response in Study 302 than was observed in Study 301 could explain the significance of Study 302” ( [4], pg. 253); and (2) “The Study 302 success could be explained by a higher placebo progression after the implementation of protocol amendment 4 while the study was ongoing” ([4], pg. 254). Protocol amendment 4 (PV4) increased dosing for ApoE carriers in the high-dose group from 6 mg/kg to 10 mg/kg. ApoE non-carriers in the high-dose group were dosed at 10 mg/kg throughout the study. (The timing of pv4 varies for each patient. Some patients were enrolled and completed the trial before pv4. Some patients were enrolled after pv4, and some patients were part way through the trial when pv4 was implemented.)

In contrast, the clinical review concluded (1) “Although the placebo decline for CDR‐SB was numerically greater in Study 302 (1.74) than Study 301 (1.56), differences in placebo response do not appear to explain why Study 302 was successful and Study 301 was not.” ([4], pg. 218).

Before evaluating the aducanumab data, it is useful to consider the 2019 FDA Draft Guidance on substantial evidence [25]: “Establishing superiority to a concurrent control group (whether an active agent, including a lower dose of the test drug, or placebo) generally provides strong evidence of effectiveness, because a superiority design does not depend on assumptions regarding the effectiveness of the control.” Although not explicitly stated in the following terms, one of the hallmarks of an adequate and well-controlled trial is that its sample size is sufficiently large so that the mean placebo response will fall within a narrow enough range that the resulting influence on the drug-placebo difference is minimal.

Studies 301 and 302 were planned in anticipation of a 2.0-point mean placebo decline on CDR-SB. This anticipation was documented in the study protocol which received SPA agreement. The mean placebo decline on the CDR-SB was 1.56 in Study 301 and 1.74 in Study 302. within the range seen in contemporary clinical trials in this patient population [26, 27], albeit less than the change assumed in study planning. This smaller-than-anticipated placebo decline could be a direct result of stopping for futility, which has a known bias towards smaller treatment effect estimates [28]. Therefore, if placebo decline influenced drug-placebo differences, it was to reduce drug-placebo differences compared to what was anticipated in study planning.

Moreover, no consistent trends were observed in placebo decline across endpoints. Compared with Study 301, Study 302 had:

- Greater mean placebo decline on CDR-SB (1.74 versus 1.56) and ADCS-ADL-MCI (− 4.3 versus − 3.8),
- Less mean placebo decline on MMSE (− 3.3 versus − 3.5), and
- Nearly identical decline on ADAS-Cog-13 (5.16 vs. 5.14)

The drug-placebo differences for low dose were similar in the two studies (− 0.18 and − 0.25). In no plausible scenario could this mean decline in the placebo groups influenced drug-placebo differences in the high-dose groups but not in the low-dose groups.

**Fig. 4:**
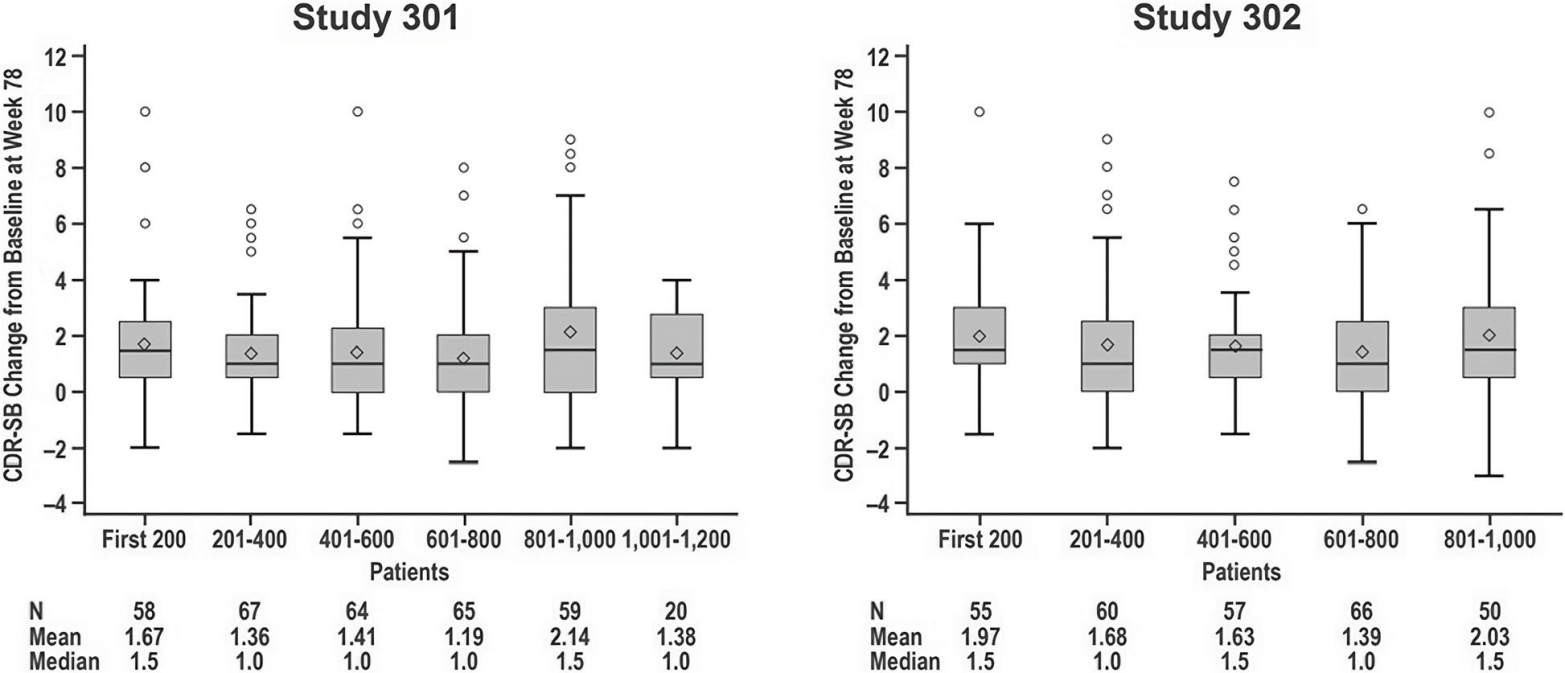
No trend in placebo decline over enrollment time (Study 301 and 302). Trends in placebo group mean changes by enrollment cohorts in Studies 301 and 302

The concern raised in the statistical review on the placebo decline pre- and post-PV4 suggests that implementation of PV4 introduced bias that caused placebo decline to change during the study. To examine this issue, the sponsor assessed placebo decline on CDR-SB in cohorts of every 200 patients enrolled. As shown in Fig. 4 ([21], Slide 33), there was no trend in placebo decline over enrollment time. The secondary endpoints also did not show any trends in placebo decline over enrollment time.

**Fig. 5:**
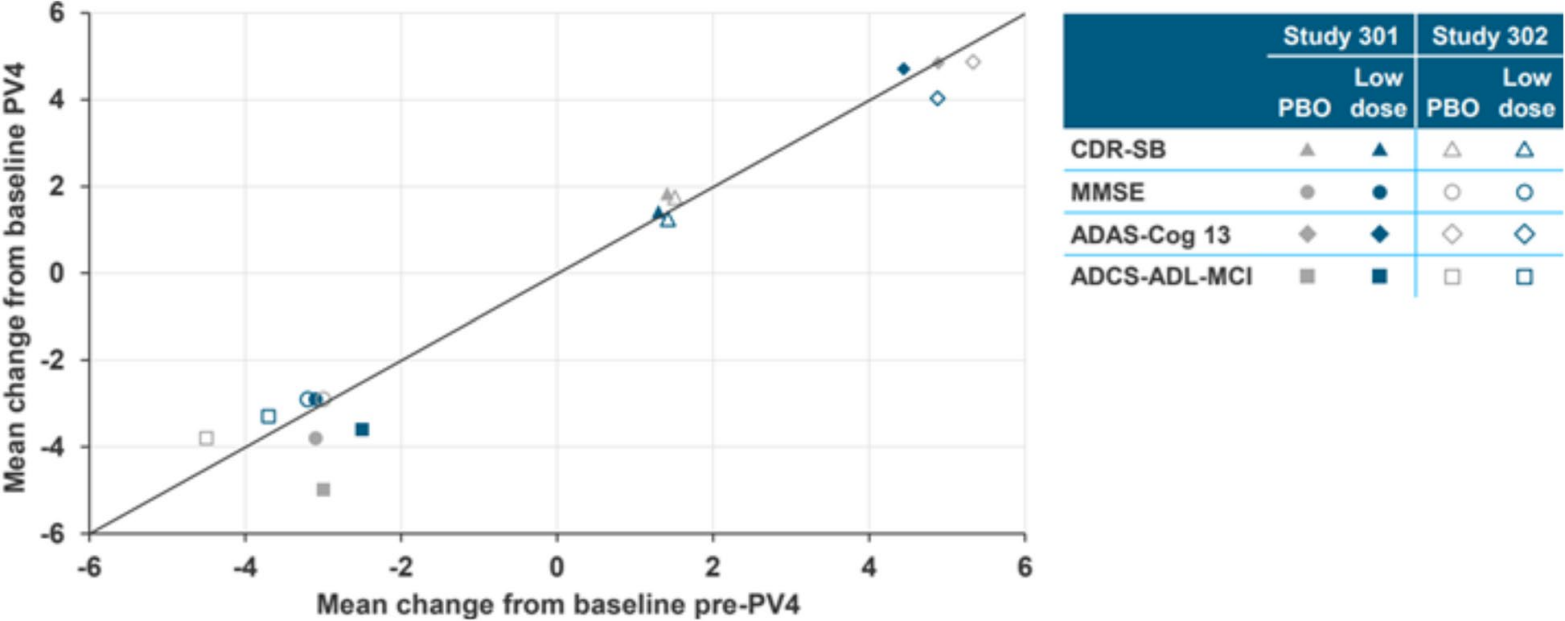
No systematic effect of PV4 in treatment arms that did not have a dose change (Study 301 and 302). Pre- and post-PV4 mean change from baseline in the placebo and low-dose groups

In addition, the sponsor compared the pre- and post-PV4 mean change from baseline in the placebo and low-dose groups. The dosing in these groups was unchanged by PV4 and therefore systematic trends in mean changes for these groups would suggest bias. The results of 16 subgroup comparisons (4 endpoints, 2 studies, 2 dose groups) for pre- versus post-PV4 are summarized in Fig. 5 ([21], slide 37).

If results were identical pre- and post-PV4, all data points would fall exactly on the line of unity. If there were no systematic trends, data points would scatter randomly around the line. If a systematic trend existed, data points would fall on one side of the line. Results show random fluctuation around the line of unity with no systematic trend, supporting the conclusion of similar placebo results pre- and post-PV4. Outcomes are in clusters because of the difference in ranges of the scales.

These results suggest (1) that mean placebo declines in Studies 301 and 302 were less than the anticipated 2.0 points on CDR-SB, potentially making it more difficult to show drug-placebo differences than anticipated in each study; (2) placebo decline did not systematically change throughout the trial; and (3) there was no indication of bias due to the implementation of PV4. These conclusions are consistent with those in the clinical review.

### Robustness of primary analysis to missing data

The statistical review questioned the validity of the primary analysis model under the missing at random assumption: (1) “There is a lot of missing data in Study 302 (and 301) at Week 78 (> 40%) caused by early stopping due to futility” ([4], pg. 270); and (2) “Given the large amount of missing data in the final ITT dataset (> 40% per group) and much lower rate missing in the Opportunity to Complete dataset, some different demographics and disease characteristics in those without the opportunity to complete (due to futility stopping) that are related to outcome and not incorporated in the primary model…, the latter OTC dataset seems more relevant and reliable.” ([4], pg. 255).

The clinical review stated: “Several SAP‐defined and post hoc sensitivity analyses were performed for the primary endpoint. The copy increment from reference method and the jump to reference method were used to test the assumption that missing data were missing at random. Results from these analyses demonstrated that the statistically significant results for the primary endpoint were not sensitive to departures from the missing at random assumption” ([4], pg. 177).

Biogen and the FDA “agreed that the dataset to be used in the final analysis of Phase 3 data would be based on all data through database lock (November 2019), with efficacy data after March 20, 2019, censored,” i.e., the ITT population( [4], pg. 38). Therefore, the primary analysis censored data collected after the futility announcement on March 20, 2019, such that the primary analysis included all the data collected under double-blind conditions. As a consequence of this censoring, it is useful to consider the two sources of missing data in Study 302: (1) administrative censoring from early termination of the trials; and (2) premature study withdrawal by individual patients.

It is reasonable to question the missing at random assumption for missing data due to administrative censoring; however, because of the well-known bias towards lower effect sizes after stopping for futility [28], data missing due to administrative censoring are more likely to favor treatment than observed data. As such, the prespecified mixed model repeated measures (MMRM) analysis, which assumes missing at random, is a reasonable but conservative primary analysis, though this censoring due to stopping for futility obviously resulted in higher missingness and lower overall power than anticipated in the study design. The SAP Addendum also prespecified supplementary analyses in which the primary analysis model would be applied to (1) the OTC dataset, which included only those patients who had the opportunity to complete the Week 78 assessment; that is, the OTC data with no administrative censoring; and (2) the ITT uncensored data in which the observations taken after the March 20, 2019, futility announcement were not censored.

The choice of dataset (ITT, OTC, ITT uncensored) had minimal influence on point estimates and did not influence conclusions because statistically significant differences were seen on CDR-SB in each dataset ([4], pg. 43).

Regarding missing data due to premature study withdrawal, the sponsor followed the guidance in the National Research Council’s expert report on the prevention and treatment of missing data [29] and ICH E9 R1 addendum [30]. Participants were encouraged to remain in the study after treatment discontinuation. These off-treatment observations were included in the primary analysis, i.e., using the ITT dataset. Robustness to departures from MAR was assessed through prespecified and post hoc sensitivity analyses. Significance was retained on all these analyses ([4], pg. 46). Therefore, concerns regarding missing data were adequately addressed and the conclusions in the clinical review were justified.

### Functional unblinding due to ARIA

The statistical review implied that Study 302 was not an adequate and well-controlled study due to functional unblinding from ARIA and quoted directly from the Guidance for Demonstrating Substantial Evidence of Effectiveness for Human Drugs and Biological Products [25], “…a randomized, double-blind, placebo-controlled trial in which unblinding is common due to an effect of the test drug, and where a modest treatment effect is found on a primary endpoint that is subject to bias when drug assignment is known (e.g., a physician global impression). In these cases, the trials might not be considered adequate and well-controlled.’ Both of these conditions are a concern in this application.” ([4], pg. 341). The clinical review concluded “… no systematic evidence of bias” ([4], pg. 232).

ARIA could lead to the perception that a participant is on active treatment. However, the incidence of ARIA in the placebo group was not trivial (10.3% in Study 302), therefore an ARIA event was not synonymous with unblinding. Moreover, safeguards in the study design and conduct were implemented to minimize the risk of functional unblinding due to ARIA. These design components were defined in the study protocols which received SPA agreement as well as global regulatory and ethics committee approvals.

Design features to minimize functional unblinding included that the management of ARIA and the administration of the clinical efficacy assessments were done by different individuals. The treating physician (usually the Principal Investigator) was responsible for the management of ARIA cases, routine neurological care, and assessment and treatment of adverse events. He/she did not have access to the post-baseline efficacy assessments. The primary efficacy endpoint (CDR-SB) was administered by an independent rater. The secondary efficacy endpoints were administered by a second independent rater. These 2 independent raters were not involved in any other aspect of participant care and management and were blinded to ARIA and other medical information. These clinical efficacy scales were also centrally reviewed by a blinded third-party vendor. Sensitivity analyses were conducted to further investigate the potential impact of functional unblinding.

One example of the sensitivity analyses is summarized in Table 1 ([4], pages 65–68). The primary analysis results for CDR-SB and the 3 secondary endpoints were compared with an otherwise identical analysis in which post-ARIA observations were removed. After excluding post-ARIA observations, treatment effects of high dose *increased* for all primary and secondary endpoints, as would be expected from the dose interruptions and reduced dosing in patients with ARIA. Results for the low-dose group showed no consistent trend. Results that are randomly distributed around the line of unity, as observed, are consistent with no bias because there is no systematic trend from excluding post-aria observations. If bias existed, the results would fall more on the side of the unity line as driven by the direction of the bias.

**Table 1:** Study 302: treatment effect at week 78, with and without post-ARIA observations excluded

	ITT population			ITT population excluding post-ARIA observations		
	Placebo	Difference vs. Placebo (%)		Placebo	Difference vs. Placebo (%)	
	Decline	Low dose	High dose	Decline	Low dose	High dose
	*N* = 548	*N* = 543	*N* = 547	*N* = 548	*N* = 543	*N* = 547
	*n* = 288	*n* = 290	*n* = 299	*n* = 254	*n* = 194	*n* = 172
CDR-SB	1.74	− 0.26 (− 15%)	− 0.39 (− 22%)	1.72	− 0.19 (− 11%)	− 0.57 (− 33%)
MMSE	− 3.3	− 0.1 (3%)	0.6 (− 18%)	− 3.4	− 0.1 (3%)	0.8 (− 24%)
ADAS-Cog 13	5.162	− 0.701 (− 14%)	− 1.400 (− 27%)	5.306	− 0.628 (− 12%)	− 2.193 (− 41%)
ADCS-ADL-MCI	− 4.3	0.7 (− 16%)	1.7 (− 40%)	− 4.3	0.5 (− 12%)	2.6 (− 60%)

*N* number of randomized and dosed patients that were included in the analysis, *n* number of randomized and dosed patients with primary endpoint assessment at week 78

Data source: 5.3.5.3 ISE_Final Table 33 and Appendix F Table 128, Table 129, and Table 130

**Fig. 6:**
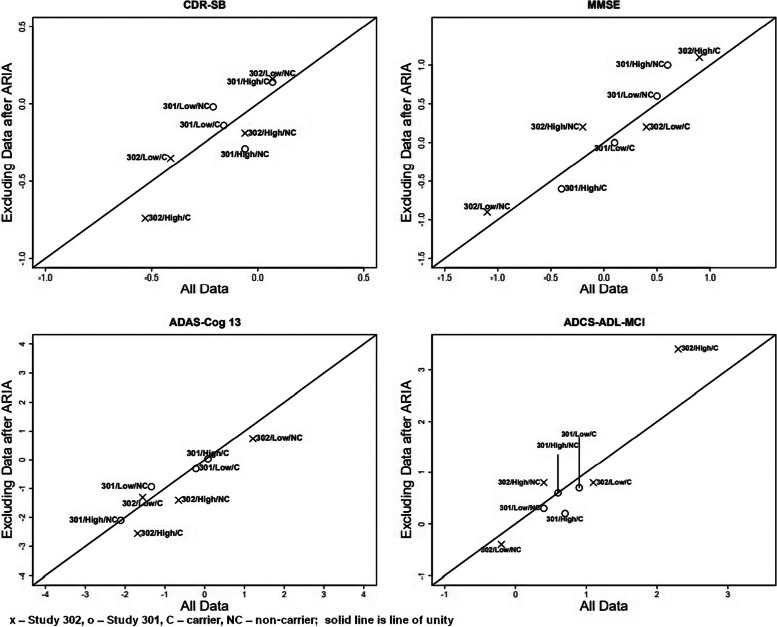
Treatment difference of change from baseline in CDR‐SB, MMSE, ADAS-Cog 13, and ADCS‐ADL-MCI at week 78 grouped by study, dose, and ApoE carrier status and including or excluding post‐ARIA observations (× – Study 302, ο – Study 301, C – carrier, NC – non‐carrier; solid line is the line of unity)

Given that ARIA incidence differs by ApoE ε4 carrier status, further analyses stratified by ApoE ε4 carrier status ([4], pg. 233) and results are summarized in Fig. 6. Each panel compares results from all data (*x*-axis) to an otherwise identical analysis with data excluded after an ARIA event (*y*-axis).

In the 8 combinations of study, treatment group, and ApoE ε4 status for each endpoint, no trend was observed comparing the treatment differences with and without post-ARIA observations. That is, the treatment differences were scattered evenly above and below the line of unity. From these analyses and review of study procedures we conclude, as did the clinical review, that the positive results in Study 302 were not an artifact of bias from functional unblinding.

### Degree of contradiction in results between Studies 301 and 302

The FDA clinical and statistical reviews disagreed as to the degree that Study 301 detracted from the Study 302 results. The statistical review stated: “We have a second large adequate well controlled study that directly contradicts the first….” ([4], pg. 253), and “… a second study which directly conflicts with the positive study” ([4], pg. 342). The clinical review states: “The results of Study 301 are sufficiently well understood that they do not preclude independent consideration of the results of Study 302 and 103” ([4], pg. 243). “Results of these exploratory analyses contribute to the overall understanding of Study 301 and together do not meaningfully detract from the persuasiveness of Study 302” ([4], pg. 245).

The following information is relevant for understanding the degree of contradiction between results from the two studies.

1. Most attention has been focused on the primary outcome of CDR-SB; however, combining evidence across the primary and secondary endpoints demonstrates directional consistency across the two studies (see Fig. 1), therefore concerns about contradiction are mostly alleviated when viewed from this perspective. Specifically, there is substantial overlap in corresponding confidence intervals for most outcomes in Study 301 and Study 302.
2. When focused on individual outcomes the following observations are also relevant:
  The low-dose group of Study 301 had results similar to the low-dose group in Study 302, with each being intermediate to the high-dose group in Study 302 ([4], pgs. 59–62);
  In Study 301, there was a significant treatment effect on β-amyloid pathology in the high-dose group. This reduction was somewhat smaller in magnitude than in Study 302, but approximately in line with the somewhat lower overall exposure in the high-dose group of Study 301;
  In Study 301, the effect for the high-dose group on CDR-SB and MMSE was similar to placebo, while the effect on ADAS-Cog13 and ADAS-ADL-MCI was similar to the low-dose groups in both studies.
  In the ITT dataset, the drug-placebo differences were 0.03 in Study 301, and − 0.39 in Study 302, for a difference between studies of 0.42. After excluding the rapid progressors, the corresponding results were − 0.09 and − 0.42, for a difference between studies of 0.33. Therefore, excluding the rapidly progressing patients (1% of the ITT dataset), accounted for one-fourth of the difference in results between studies.
  Extensive data mining was devoted to understanding the effects of dosing on the difference in study results, and this extensive review failed to explain these differences. Full accounting of the results goes beyond the present scope. However, the clinical review concluded that dosing differences between the two studies had a small contribution to the divergent results in the high-dose arms ([22], slide 29).

Therefore, the overall interpretation is in agreement with the clinical review in that results of the two studies are partially discordant. However, because the results of the high-dose arm were contradictory, the data do not support the conclusion that the Study 301 high-dose results do not detract from Study 302 high-dose results. Given less than one-half of the difference between studies was explained, perhaps only one-third, the high-dose arm results of Study 301 must, to some degree, offset the positive results from Study 302. Figure 1 shows how much Study 301 detracts, by comparing the GST results for Study 302 alone (*p* = 0.0001) to the combined GST results at the bottom of the table (*p* = 0.0003).

## Discussion

Much has been said regarding the unusual circumstances leading to the approval of aducanumab. Part of that discussion was fueled by differences in interpretations between the FDA clinical and statistical reviews. However, little attention was given to why those opinions differed. The present examination of the publicly available aducanumab data showed that in most of the key instances the statistical review was flawed and the conclusions were incorrect. These errors influenced Advisory Committee member votes and these mistakes were further propagated in scientific journals [5, 6] and in the media.

Although the conclusion from the clinical review that Study 302 was an especially persuasive result was justified, the clinical review went too far in concluding that results from Study 301 did not detract from the Study 302 results. Only part of the difference in results between the high-dose arms was explained via the extensive post hoc analyses. The unexplained part should not have been disregarded.

These findings have important implications for ongoing and future studies. The divergence in results between the high-dose arms was real, although neither study was flawed, with the exception of stopping early for futility. Therefore, it is likely that results from other large, well-designed studies could show similar divergence, particularly if they stop early for either futility or efficacy. As has already been seen with aducanumab, this degree of heterogeneity leads to problematic interpretations of confirmatory studies and also has serious implications for smaller studies.

The aducanumab results are generally representative of similarly sized AD studies. One study showed a 0.28-point advantage over placebo on the primary outcome of CDR-SB while the twin study showed a 0.22-point difference in the opposite direction ([22], slide 6). That is, the difference in results between studies was 0.50 points on the CDR-SB, the magnitude of treatment effect the full studies were powered to detect. With this degree of divergence in identically designed studies, it is difficult to be confident in individual proof-of-concept studies whether the results are positive or negative if we are solely relying on a single outcome.

Returning to the three major challenges in AD research: accurate diagnosis, accurate measurement of disease severity, and reliable analysis of the data, the aducanumab results point to the need for improvement on the latter two, while not precluding need for improvement on the first.

For example, imbalances in the small number of rapidly progressing patients meaningfully influenced aducanumab results, which were based on an MMRM analysis. Methods such as robust regression and non-parametric analyses buffer the effects of unusual observations and may be better alternatives than MMRM when data are heavily skewed as were the aducanumab data and as may be expected in degenerative diseases in general. Stopping the studies early likely exaggerated these effects since Study 301 had a particularly negative result at the time the study was stopped.

In addition, consider the large disparity in slowing of decline versus placebo across endpoints within studies (20% difference between best and worst results in Study 301 and 22% difference in Study 302). This disparity in established endpoints suggests that optimized item combinations of selected items within and across scales may be needed, thereby leading to more consistent and precise estimates of drug effects. The discordance in aducanumab results also suggests potential benefit from other ways to synthesize results across endpoints and studies, such as the GST approach shown above. However, more research is needed to understand the merits and optimal implementation of alternative assessments and analyses.

## Conclusions

The statistical review of the aducanumab data was incorrect in a number of key inferences and these mistakes were propagated in subsequent publications and media stories. The clinical review went too far in saying the Study 301 results did not meaningfully detract from the exceptionally persuasive results in Study 302, though it generally did demonstrate a more defensible statistical approach than the statistical review itself. Much of the controversy surrounding the aducanumab review and subsequent approval were likely driven by the large discrepancies between the statistical and clinical reviews, each of which gained traction with audiences whose perspectives aligned with their conclusions. While we can’t know for certain how much communication occurred between the two teams, the clinical review referenced and acknowledged the statistical review, while the statistical review appeared more siloed in nature, and interestingly, is still labeled as draft.

Many things contributed to the controversy in this situation. It started with the inconsistency between stopping for futility and later announcing positive results (partly a result of a major protocol amendment). Divergent results between the two studies on the primary outcome contributed to the confusion. Differences in the FDA reviews added to the controversy which was then magnified with the Advisory Committee meeting and the media follow-up. Additional analyses were performed by the FDA to address the concerns, but by this point, parties with different understandings of the issues ceased trying to understand all sides of the issue. This process could have been stopped at any stage, but incorporating the secondary endpoints into the interpretation through a GST or other evaluation would likely have avoided the futility decision and would have reduced the apparent divergence of the study results.

Ultimately, the conclusion on whether the treatment effect is real will depend on the reader’s perspective on the required level of evidence, potential multiplicity corrections, and the broader context of the study. We believe the results are statistically compelling, but clinical meaningfulness is also an important consideration and is beyond the scope of this discussion. It is time to move past the aducanumab controversy and focus research on methods to minimize the likelihood of similarly divergent results in future studies.

## Supplementary Information

**Additional file 1: Supplemental Material.** Two Letters to the Advisory Committee and FDA from the authors

## Data Availability

All data accessed was publicly available and is published in sources referenced, particularly the original study publication^1^ and the advisory committee briefing document^4^.
