# Supplementary material for: Avoiding future controversies in the Alzheimer’s disease space through understanding the aducanumab data and FDA review
Source: Alzheimers Res Ther. 2023 May 24;15:98. doi: 10.1186/s13195-023-01238-1 (PMC10207692; doi:10.1186/s13195-023-01238-1)
Supplement: Supplementary file 1 — Additional file 1: Supplemental Material. Two Letters to the Advisory Committee and FDA from the authors [file 13195_2023_1238_MOESM1_ESM.docx]

Supplementary Material – Two Letters to the Advisory Committee and FDA from the authors

FDA Letter #1 – posted Nov 3, 2020

Comment ID

FDA-2018-N-0410-0042

Dear FDA and Committee Members (Docket No. FDA-2018-N-0410),
Statistical evidence is strong for a treatment benefit in the Aducanumb ENGAGE and EMERGE studies, contrary to what is generally believed, although statistical interpretation is not the only consideration. Alzheimer’s disease is a complex, multifactorial disease with outcomes that represent various symptoms of disease, but no single measure that encompasses the total disease state. This often results in divergent effects across primary and secondary outcomes in clinical studies. The core symptoms of clinical progression in Alzheimer’s disease fall into cognitive, functional and global domains. Cognition is usually a performance measure of the participant, function is measured by activities of daily living reported by a study partner, and global assessments are performed by clinicians based on interviews with the participant and study partner. In this population, these are usually measured with the ADAS-cog, ADCS-ADL and CDR-sb, which were primary and secondary outcomes in the ENGAGE and EMERGE studies.
If there were one clear way to measure Alzheimer’s disease, this measurement would be the gold standard for AD trials. Without this clear gold standard, we must rely on separate measures of symptoms, all of which are driven by one underlying disease process. A combined outcome across these mostly independent domains is a close approximation to this gold standard. We used a global statistical test (GST) to calculate this combined outcome (GST) for the ENGAGE and EMERGE studies based on publicly presented data (Table 1). The estimated p-values and effect sizes in ADAS-cog, ADCS-ADL and CDR-sb are also shown to help in assessing consistency across the outcomes.
The overall level of evidence (GST) across the ENGAGE study has a 2-sided p-value of 0.2817 with an effect size of 9.1%, while the EMERGE study has a 2-sided p-value of 0.0001 with an overall effect size of 30.3%. Both studies favor Aducanumab. The combined GST across the two studies resulted in a p-value of 0.0003 with an effect size of approximately 20%, suggesting that the two studies combined have slightly less evidence for a treatment effect than the EMERGE study alone, but the combined effect size is reduced by 33% compared to EMERGE alone. Statistically, the ENGAGE results do not strengthen nor weaken, and certainly do not invalidate the evidence from the EMERGE study.
There are several outstanding questions that are not answered by publicly available data, such as: Is the effect similar in APOE-e4 positives and negatives? Is the effect driven by a few sites? Are effects consistent across milder and less mild participants? Are the safety issues manageable? Is the risk-benefit acceptable? Has the regulatory standard been met? Can a very positive study carry a directionally positive but inconclusive study? Is another study needed?
While the statistical support appears strong, statistical considerations are only one aspect of the decision-making process in evaluating the Aducanumab data. Some of the issues mentioned above will likely be topics of discussion at the Advisory Committee meeting. Given these complexities, the appropriate outcome of the meeting – approval, approval with commitments, or rejection -- is uncertain without the additional information that will be available to the FDA and the Advisory committee.
Thank you for the opportunity to comment.
Pentara Corporation Statisticians
Suzanne Hendrix, PhD
Sam Dickson, PhD
Sean Hennessey, MS
Jessie Nicodemus-Johnson, PhD
Newman Knowlton, MS
Logan Kowallis, PhD

NOTE: The calculations in this letter are based on publicly presented figures and numbers and are not as accurate as they could be with access to the raw data. The p-values and effect sizes are shown with more precision than we have access to, in order to allow comparison between numbers. The conclusions regarding level of evidence would not be expected to change substantially with access to raw data. Pentara provides consulting for over 30 pharmaceutical, biotech, non-profit and academic groups in the neurodegenerative space but had no involvement in the Aducanumab studies.

FDA Letter #1 – Attachment 1


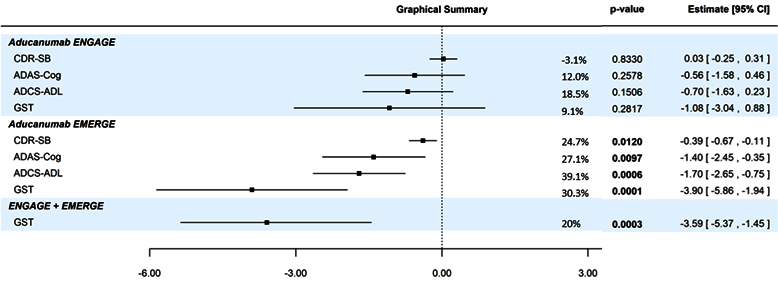


FDA Letter #2 - Attachment 2

Many points have been raised that are worth further discussion in this context:

1. Since the original studies stopped for futility, isn’t it all over now? Is it justified to make conclusions from a failed study?

*Stopping for futility, or even testing whether you should stop for futility can’t inflate the type 1 error (alpha) since type 1 error is the probability of declaring a study successful by chance alone. Declaring futility can’t increase your chance of declaring success. The alpha from the original hypothesis for both studies is still intact at a 2-sided alpha of 0.05.*

1. Didn’t stopping the studies early introduce bias that makes it impossible to make any conclusions?

*The original hypothesis can still be tested with the data that is available. By stopping the study early, there are many missing data points, but they are missing systematically from both the active and the placebo groups, so they are less likely to introduce bias in the estimated treatment effects.*

*However, the protocol amendment that allowed the 10mg dose for the APOE-e4 positive participants resulted in a higher average dose exposure in the EMERGE study since it was started later with more patients enrolling after the amendment. This suggests that the two studies are likely measuring the treatment effect of different dose exposures, potentially resulting in an underestimate of the 10mg treatment effect in the ENGAGE study, and more accurate estimate for the 10mg treatment effect in the EMERGE study, if there is a true dose response. These details will need to be investigated by the FDA and the Advisory Committee.*

1. Isn’t this just another post-hoc analysis trying to get something positive out of failed studies?

*Yes, it is post-hoc, but it is the closest analysis for answering the primary hypothesis question, which is “Does this treatment slow down the progression of Alzheimer’s disease?” Use of the CDR-sb as a primary endpoint is an FDA accepted endpoint in this stage of disease, but it isn’t the best measure of the overall progression if we’re assessing the totality of disease.*

1. Is it reasonable to use the GST outcome instead of the pre-specified primary endpoint (CDR-sb)?

*The GST analysis is intended to answer the broader question of whether Aducanumab slows Alzheimer’s disease by combining evidence from primary and secondary outcomes. Interestingly, the CDR-sb results from ENGAGE and EMERGE, combined in a GST across studies has a p-value of 0.007 and an effect size of approximately 11% slowing of progression despite the ENGAGE results being slightly in favor of placebo. So, the primary results and the GST results across both studies show strong evidence for a treatment effect.*

1. Are the subgroup analyses from ENGAGE supportive of the EMERGE results?

*Post-hoc subgroup analyses are always problematic if they are being relied on for demonstrating primary efficacy, so the subgroup analyses from the ENGAGE are not useful in this regard. Once statistical efficacy is established, they can be useful for assessing which groups are driving that efficacy.*

1. What about the lack of correlation between the biomarkers and the clinical outcomes?

*Amyloid biomarkers assess target engagement and should not be expected to serve as clinical surrogate marker of disease progression. Generally, lack of biomarker evidence shouldn’t be used to invalidate a clinical effect unless the biomarker has been established as a surrogate.*

1. Haven’t all the studies testing the amyloid hypothesis conclusively failed?

*There is evidence of efficacy in other amyloid studies when looking at the totality of evidence. For instance, the combined evidence across the ADAS-cog, CDR-sb and ADCS-ADL in the Expedition 3 study results in a p-value of 0.0011 and a 13.8% slowing of disease progression.*

1. Didn’t the ARIA events in the high-dose group cause unblinding that would fully explain the apparent treatment benefit?

*Unblinding is more likely to affect subjective measures (ADCS-ADL and CDR-sb), not cognitive performance measures (ADAS-cog). The ADAS-cog effect across both studies has a p-value of 0.0013 and an effect size of approximately 20% slowing, which is almost identical to the results using the overall GST combined across both studies and all 3 outcomes, suggesting that any potential unblinding is not likely to be responsible for the treatment effect. The ADAS-cog effect was partway between the CDR-sb effect (clinician rating) and the ADCS-ADL effect (caregiver assessment), both of which could have been influenced if there was unblinding. Since they do not show consistent direction relative to the ADAS-cog, it is unlikely that unblinding is responsible for the treatment effect.*

FDA Letter #2 – posted Nov 9, 2020

Comment ID

FDA-2018-N-0410-0055

Dear FDA and Committee Members (Docket No. FDA-2018-N-0410),

Several legitimate concerns were raised by the statistical reviewer that are still unanswered based on available information:

1. The faster placebo decline that was observed in the ENGAGE 301 study after the protocol amendment
2. The country differences
3. The larger effect in mild AD compared to MCI
4. The apparently divergent effect between APOE-e4 carriers and non-carriers

While the statistical review presented a complete picture for several results for both the primary and all of the secondary endpoints in order to get a complete picture of the support for efficacy. This approach should have been carried over to the subgroup analyses such as APOE e4 positive vs negative, countries, MCI vs mild AD (or severity of disease) and before and after the PV4 amendment. The correlations between primary and secondary were cited as ranging between 0.40 - 0.70 and suggested to be nearly interchangeable, however subtracting off the proportion of overlapping information (square the correlation) leaves between 51% and 84% of independent variation. This suggests that a combined interpretation with both primary and secondary variables would be more informative.

The divergent outcomes that are seen with multiple imputation and across many of the subgroups are likely a result of the high variability that is frequently observed in Alzheimer's clinical studies. This high within and between patient variability is largely responsible for late stage failure in this field. The GST approach that we described in our previous letter allows stabilization of the variability and would likely lead to a more consistent result -- positive or negative.

Because of the small sample sizes within these subgroups, the added stability afforded by the GST method would allow more conclusive interpretation of these issues. Without it, it is not possible to fully understand this data.

Also, we agree with the statistical reviewer that the PRIME study results should not be used as support for the efficacy of Aducanumab due to the escalating dose design and the fact that only a fraction of the placebo group was concurrent with the 10 mg/kg dose arm. As stated in our earlier letter, we still believe that the correlative analyses between the biomarkers and the efficacy outcomes are not appropriate unless the biomarkers are progressive surrogates of disease in the relevant population, which they are not.

Thank you for the opportunity to comment,

Suzanne Hendrix, PhD
Jessie Nicodemus Johnson, PhD

NOTE: The MMSE is usually used as a screening tool and in clinical practice and has never been used as an acceptable regulatory endpoint in AD and should not be used to evaluate the overall treatment effect. The cognitive domain is better represented by the ADAS-cog which is an acceptable regulatory endpoint for cognition.
